# The impact of serological features in Chinese children with primary or past Epstein–Barr virus infections

**DOI:** 10.1186/1743-422X-10-55

**Published:** 2013-02-13

**Authors:** Yuan Huang, Cong Wei, Kun Zheng, Dongchi Zhao

**Affiliations:** 1 Pediatrics Department, Zhongnan Hospital, Wuhan University, 430071, Wuhan, China; 2 Pediatrics Department, Children’s Hospital, Zhejiang University School of Medicine, 310000, Hangzhou, China

**Keywords:** Epstein–Barr virus, Children, Primary or post infection

## Abstract

**Background:**

Epstein–Barr virus (EBV) is a primary cause of infectious mononucleosis (IM) throughout the world, and the positive serology rate changes over time in infected individuals. The aim of this study was to explore the serological and clinical features among Chinese children with EBV infections. A retrospective study of children suspected of having IM was conducted. Peripheral blood samples were analyzed by indirect immunofluorescence to detect any EBV-specific antibodies. Samples were classed as positive (+) or negative (−) to immunoglobulins M (IgM) or G (IgG) to the viral capsid antigen (VCA) or EBV nuclear antigen (EBNA). A standard medical history was taken, including epidemiological data and noting any clinical manifestations.

**Results:**

Of 317 children, 37 were aged <8 months; 10 of these were VCA-IgM+, and the youngest was aged 1 month; 280 were aged >8 months. The EBV infection rate ranged from 21.4% among subjects aged 8–12 months to 84.2% in those aged >9 years. Serologically, children who tested as VCA-IgM+ together with VCA-IgG and EBNA-IgG– had longer hospital stays with more palatal petechiae and lymphadenopathy, especially among those with an atypical lymphocyte count of >10%. Children with the serological patterns [VCA-IgM–, VCA-IgG+ and EBNA-IgG–] and [VCA-IgM+ VCA-IgG+ and EBNA-IgG+] did not show specific clinical features.

**Conclusions:**

Infants aged <8 months could be infected with EBV. About 84% of these Chinese children aged >9 years had serological evidence of EBV infection, whereas IM peaked in patients aged 2–3 years.

## Background

Epstein–Barr virus (EBV), a member of the herpesvirus family, is one of the primary causes of infectious mononucleosis (IM) in children and teenagers, and is widespread throughout the world. Following primary infection, the virus establishes lifelong latency, and more than 90% of adults today have serological evidence of past infections [1,2].

The indirect immunofluorescence assay (IFA) is widely used for the routine evaluation of EBV immunological status [3-5]. Although the EBV genome encodes many structural and nonstructural genes, those of most importance for serodiagnosis are the genes encoding the viral capsid antigens (VCAs), the early antigens (EAs) and the virus nuclear antigens (EBNAs). Four serological parameters are essential for detecting EBV-specific immunoglobulin M (IgM) or immunoglobulin G (IgG) antibodies in immunocompetent individuals on a qualitative basis: IgM-VCA, VCA-IgG, EA-IgG and EBNA-IgG. VCA-IgM is generally designated as an indicator of a recent primary infection [2]. Nevertheless, VCA-IgM might appear later, or might be produced only transiently, or might persist at such a low concentration as to be missed by laboratory tests and the low and high affinity IgG have different significance during EBV infection, low affinity anti-VCA IgG suggests a early stage infection and high anti-VCA IgG suggests post infection[6]. In addition, some patients do not produce EBNA-1-IgG [7], and even if this antibody is generated, it can disappear, especially among patients with immunosuppression [8,9]. Therefore, the routine addition of an estimation of anti-VCA-IgG avidity to diagnostic EBV serology is recommended, and the combination of EBNA-IgG– and low-avidity VCA-IgG+ has excellent sensitivity and specificity [4,10].

EBV seropositivity among children has much geographic variation. The positive rate is much higher among children in Asia than those in Western countries [11-14]. This study aimed to explore serological outcomes and clinical features in children with EBV infection and to interpret the implications.

## Methods

A total of 317 inpatients (197 boys and 120 girls), with ages ranging from 1 to 164 months, were enrolled for this retrospective study. All were admitted to Zhongnan Hospital of Wuhan University, P. R. China, between July 2008 and August 2010, suspected of having IM. They had one of the following signs: (1) at least three of the EBV-related symptoms of fever, rash, lymphadenopathy, pharyngitis, palatal petechiae, hepatomegaly, or splenomegaly; (2) fever lasting longer than five days; (3) respiratory tract infection symptoms lasting longer than five days and unresponsive to conventional antibiotic treatment. Children with EBV-associated malignant diseases such as malignant lymphoma or chronic active EBV infection were excluded.

Informed consent was obtained from each patient parent at the time that serum samples were collected for assessing EBV antibody status. The study was approved by the Ethics Committee of Wuhan University Zhongnan Hospital, in accordance with the Helsinki Declaration.

### Definition of EBV infection

A primary infection was defined as the presence of VCA-IgM, or positive assays for EA-IgG or low-affinity anti-VCA-IgG. Past infection was defined as a positive assay for IgG to VCA and IgG to EBNA, or the detection of high-affinity anti-VCA-IgG without VCA-IgM or EA-IgG. Uninfected children were defined as having no detectable antibodies to EBV. Peripheral blood samples were obtained from all children within 24 h after admission to the pediatric department. Specific antibodies to EBV were detected using a commercial indirect immunofluorescence (IIF) kit IFA (EUROIMMUN, Lübeck, Germany) following the manufacturer’s instructions [10,15,16]. The diagnosis of IM based on Sumaya criteria [17].

### Statistical analysis

Data are presented as the percentage or mean ± standard deviation (SD). All statistical analyses were performed using SPSS software (version 16; SPSS Inc., Chicago, IL, USA). The chi-squared test was used to compare between-group differences in percentages. The rank–sum test was used to compare differences between the dates of measurements, and P *<* 0.05 was accepted as statistically significant.

## Results

### Serological features of EBV antibodies

Of 317 patients, 37 children were aged <8 months: 27 boys and 10 girls. The EBV antibody patterns are shown in Table 1. The serological pattern [VCA-IgM–, VCA-IgG and EBNA-IgG+] was the most common combination. Three patients aged 5, 6 and 7 months had the pattern [VCA-IgM+, VCA-IgG and EBNA-IgG–]. The youngest child with a primary infection was only 1 month old. There were 280 children aged ≥8 months: 170 boys and 110 girls. The seropositive patterns included 95 (33.9%) with a past infection, 60 (21.4%) with a primary infection and 9 (3.2%) with orphaned VCA-IgG+ (Table 2).

**Table 1:** EBV antibody combinations in EBV-positive patients aged <8 months (n = 37)

**VCA-IgM**	**Low affinity**	**VCA-IgG**	**EBNA-IgG**	**EA-IgG**	**n**	**Percentage (%)**	**Infection status**
+	–	–	–	–	3	8.1	Primary infection
+	–	–	+	–	2	5.4	Primary infection
+	+	–	+	–	1	2.7	Primary infection
+	+	+	+	+	1	2.7	Primary infection
–	+	+	+	–	1	2.7	Primary infection
–	+	+	–	+	1	2.7	Primary infection
–	–	+	+	+	1	2.7	Past infection or maternal antibodies
–	–	+	+	–	10	27.0	Past infection or maternal antibodies
–	–	+	–	–	1	2.7	Past infection or maternal antibodies
–	–	–	+	–	2	5.4	Past infection or maternal antibodies
–	–	–	–	–	14	37.8	No infection

**Table 2:** EBV antibody combinations in EBV-positive patients aged ≥8 months (n = 280)

**VCA-IgM**	**Low affinity**	**EA-IgG**	**VCA-IgG**	**EBNA-IgG**	**N**	**Percentage (%)**	**Infection status**
+	–	–	–	–	7	2.5	Primary infection
+	–	–	+	+	8	2.9	Primary infection
+	–	–	+	–	1	0.4	Primary infection
+	–	–	–	+	1	0.4	Primary infection
+	–	+	–	–	1	0.4	Primary infection
+	–	+	–	+	1	0.4	Primary infection
+	+	–	–	–	6	2.1	Primary infection
+	+	+	–	–	3	1.1	Primary infection
+	+	+	+	–	1	0.4	Primary infection
+	+	–	+	–	1	0.4	Primary infection
–	+	–	–	–	1	0.4	Primary infection
–	+	–	+	–	2	0.7	Primary infection
–	+	+	+	–	1	0.4	Primary infection
–	–	+	–	–	4	1.4	Primary infection
–	–	+	+	–	1	0.4	Primary infection
–	–	+	+	+	21	7.5	Primary infection
–	–	–	+	+	91	32.5	Past infection
–	–	–	–	+	4	1.4	Past infection
–	–	–	+	–	9	3.2	Orphaned VCA-IgG
–	–	–	–	–	116	41.4	No infection

**Figure 1:**
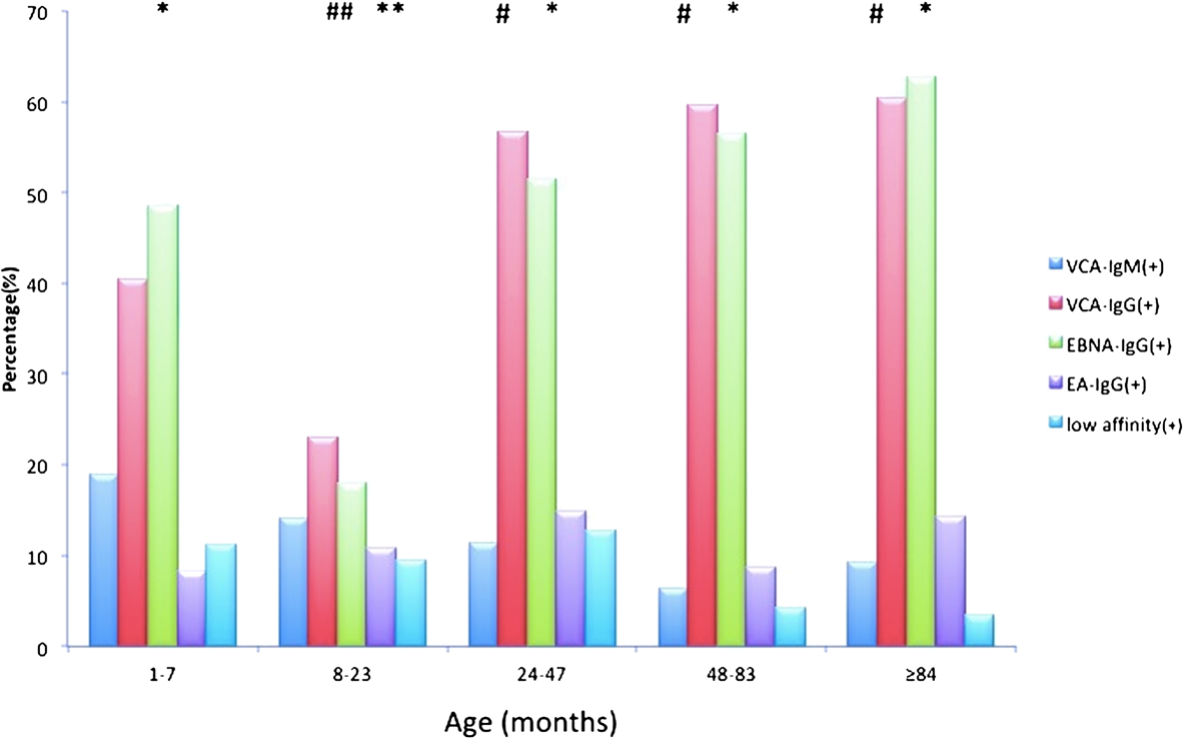
**The distribution of antibodies in EBV-positive patients.***P* < 0.05 between * and **, and between ^#^ and ^##^.

The distribution of antibodies by age is presented in Figure 1. The trends in the age distribution of VCA-IgG and EBNA-IgG antibodies were similar. The detection rates were the lowest among children aged 8–24 months and increased gradually with age. The detection rates of VCA-IgG and EBNA-IgG reached 60.5% and 62.8%, respectively, in children aged ≥7 years. Patients aged 8–24 months had a lower detection rate of EBNA-IgG than the other four groups (*P* < 0.001). Compared with the age groups of 2–4 years, 4–7 years and ≥7 years, the patients aged 8–24 months had the lowest detection rates for VCA-IgG (*P* < 0.001).

### EBV seropositivity rate by age

**Figure 2:**
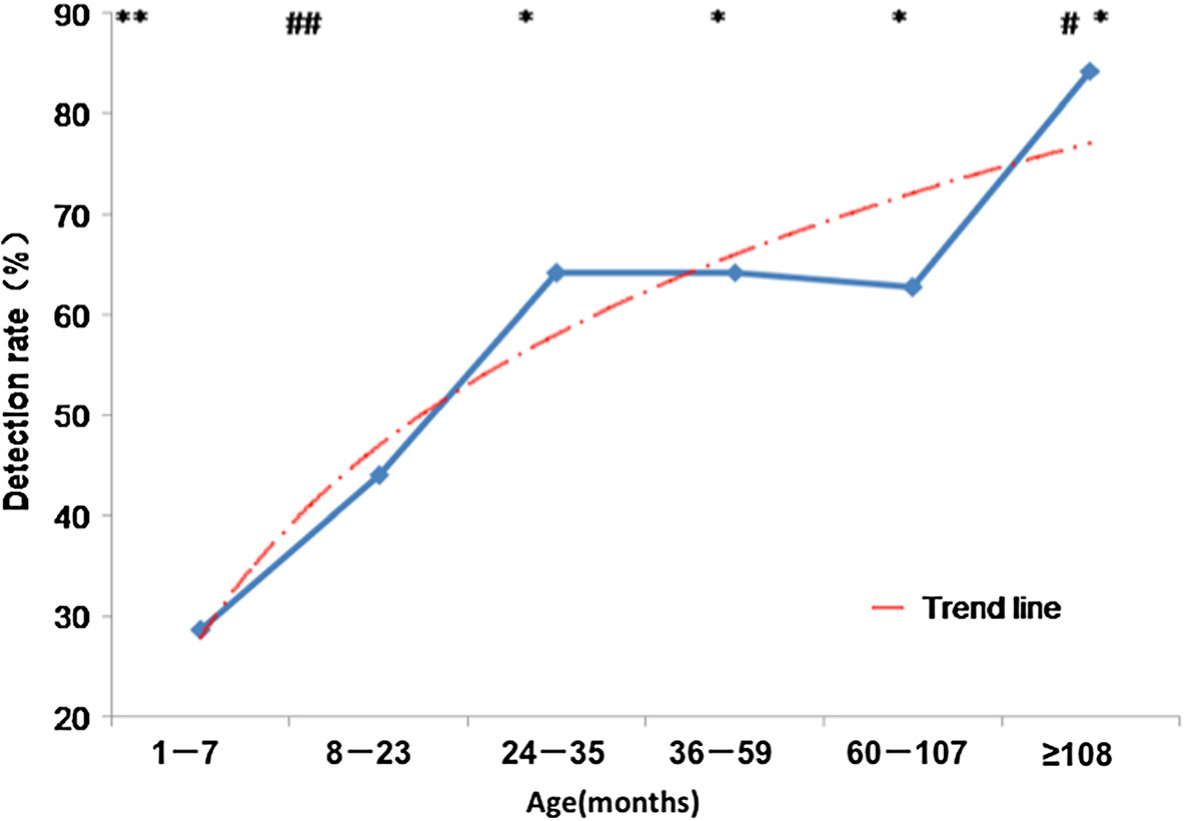
**EBV infection rates by age.***P* < 0.05 between * and **, and between ^#^ and ^##^.

The patients were classified into five age groups. The presence of at least one positive result for VCA-IgM, VCA-IgG or EBNA-IgG indicated that they had been infected with EBV. The seropositive rate increased among children aged 8–36 months and then plateaued among children aged 36–108 months. For children aged ≥9 years, the positive rate was 84.2% as shown in the trend line (Figure 2).

### The disease spectrum of EBV infection

The EBV-related diseases were diverse (Table 3). The most common disease caused by a primary infection was respiratory tract infection (41.7%), followed by IM (40.0%), Kawasaki disease, anaphylactic purpura, idiopathic thrombocytopenic purpura, measles and asthma. Seven of the nine patients presenting with orphaned VCA-IgG were diagnosed with a respiratory tract infection.

**Table 3:** The disease spectrum in EBV-positive children

**Diagnosis**	**Primary infection**	**Past infection**	**Orphaned VCA-IgG**	**No infection**
	**(n = 60)**	**(n = 95)**	**(n = 9)**	**(n = 116)**
IM	24 (40.0%)	3 (3.2%)	1 (14.3%)	5 (4.3%)
Respiratory infection	25 (41.7%)	74 (77.9%)	7 (77.8%)	94 (81.0%)
Kawasaki disease	2 (3.3%)	6 (6.3%)		7 (6.0%)
ITP	1 (1.7%)	1 (1.1%)		
Anaphylactic purpura	1 (1.7%)	1 (1.1%)		
Ulcerative stomatitis	1 (1.7%)			
Measles	1 (1.7%)			1 (0.9%)
Asthma	1 (1.7%)	2 (2.1%)		3 (2.6%)
JRA	1 (1.7%)	1 (1.1%)		
Pulmonary tuberculosis	1 (1.7%)	1 (1.1%)		
Herpangina	1 (1.7%)	1 (1.1%)		2 (1.7%)
Hand-foot-mouth disease	1 (1.7%)			
Summer fever		1 (1.1%)	1 (14.3%)	
GERD		1 (1.1%)		
CMV infection		1 (1.1%)		
Virus infection		1 (1.1%)		1 (0.9%)
Fever of unknown origin		1 (1.1%)		
Exanthema subitum				1 (0.9%)
Adenolymphitis				1 (0.9%)
Blood poisoning				1 (0.9%)

**IM**, infectious mononucleosis; **ITP**, idiopathic thrombocytopenic purpura; **JRA**, juvenile rheumatoid arthritis; **GERD**, gastroesophageal reflux disease.

### EBV primary/past infections and clinical features

To analyze the clinical features of EBV infection by age, patients aged ≥8 months were classified into four groups: 60 had a primary EBV infection, 95 had a past infection, nine had orphaned VCA-IgG and 116 were uninfected. The distribution in these four groups by age is shown in Table 4. The age group of 1–2 years had the highest detection rate for a primary EBV infection. The incidence of past infection increased gradually with age from 3.6% to 46.8%, and the patients aged 8–12 months had a lower incidence than the other three groups (*P* < 0.001) [Table 5]. These results indicate that EBV primary infection could be detected in all age groups.

**Table 4:** The distribution of age in EBV-positive patients (age ≥8 months)

**Infection status**	**Age (months)**				
	**8–11**	**12–23**	**24–35**	**36–59**	**≥60**
Primary infection (n = 60)	6 (21.4%)	13 (26.0%)	13 (24.5%)	13 (18.6%)	15 (19.0%)
Past infection (n = 95)	1 (3.6%)**	7 (14.0%)^##^	19 (35.9%)*	31 (44.3%)*^#^	37 (46.8%)*^#^
Orphaned VCA-IgG (n = 9)	1 (3.6%)	2 (4.0%)	2 (3.8%)	2 (2.9%)	2 (2.5%)
No infection (n = 116)	20 (71.4%)**	28 (56.0%)	19 (35.9%)*	24 (34.3%)*	25 (31.7%)*
Total	28	50	53	70	79

*P* < 0.05 between * and **, and between ^#^ and ^##^.

**Table 5:** The distribution of EBV primary-infected patients by age

**Primary infection (n = 69)**	**Age (months)**		
	**1–23**	**24–35**	**≥36**
IM (n = 24)	7 (25.0%)	7 (53.9%)	10 (35.7%)
Non-IM (n = 45)	21 (75.0%)	6 (46.1%)	18 (64.3%)
Total	28	13	28

There were no differences between these age groups in clinical features, but the mean hospital stay in the primary-infected group was longer than in the past-infected group (*P* < 0.001). The age distribution of children with a primary EBV infection is shown in Table 6. The detection rate of IM was highest among children aged 2–3 years. The clinical features in patients with a primary EBV infection divided into those with and without IM are listed in Table 7. Compared with the non-IM group, the IM group with a primary EBV infection had more frequent presentations of lymphadenopathy, pharyngitis, palatal petechiae, abnormal chest X rays, hepatomegaly and splenomegaly, an atypical lymphocyte count >10% and abnormal liver function. Patients without IM were classified into three groups: 36 had a primary HBV infection, 92 had a past infection and 111 were uninfected (Table 7). Rash was more frequent in children with a primary HBV infection than in the other two groups (*P* < 0.01), but they had a lower rate of abnormal chest X rays (*P* < 0.001).

**Table 6:** Features in patients with an EBV primary infection

**Clinical features**	**Primary infection**	
	**IM (n = 24)**	**Non-IM (n = 36)**
Age (months)	47.8 ± 38.8	41.8 ± 32.3
Male/female patients	11/13	17/19
Length of stay (days)	11.0 ± 2.7*	9.1 ± 4.1
Fever	23 (95.8%)	31 (86.1%)
Cough	12 (50.0%)*	30 (83.3%)
Rash	4 (16.7%)	14 (38.9%)
Lymphadenopathy	20 (83.3%)*	11 (30. 6%)
Pharyngitis	23 (95.8%)*	26 (72.2%)
Palatal petechiae	13 (54.2%)*	3 (8.3%)
Abnormal chest X ray	6/7 (85.7%)*	7/20 (35.0%)
Hepatomegaly	11 (45.8%)*	0 (0.0%)
Splenomegaly	8 (33.3%)*	0 (0.0%)
ALC >10%	17/23 (73.9%)*	3/21 (14.3%)
Elevated ESR	11/20 (55.0%)	10/21 (47.6%)
Elevated CRP	9/19 (47.4%)	10/21 (47.6%)
ALF	13/18 (72.2%)*	4/18 (22.2%)

**IM**, infectious mononucleosis; **Non-IM**, patients without IM; **ALC**, atypical lymphocytes; **ESR**, erythrocyte sedimentation rate; **CRP**, C-reactive protein; **ALF**, abnormal liver function (alanine aminotransferase or aspartate aminotransferase levels >46 U/L). *P < 0.05 versus the non-IM group.

**Table 7:** Features in patients not diagnosed with IM

**Clinical features**	**Primary infection(n = 36)**	**Past infection(n = 92)**	**No infection(n = 111)**
Age (months)	41.8 ± 32.3*	60.3 ± 37.7**	37.7 ± 29.5*
Male/female patients	17/19	57/35	74/37
Length of stay (days)	9.1 ± 4.1	7.8 ± 2.5*	8.9 ± 3.9
Fever	31 (86.1%)	83 (90.2%)	97 (87.4%)
Cough	30 (83.3%)	65 (70.6%)	85 (76.6%)
Rash	14 (38.9%)**	17 (18.5%)*	16 (14.4%)*
Lymphadenopathy	11 (30.6%)	33 (35.9%)	29 (1.8%)*
Pharyngitis	26 (72.2%)	74 (80.4%)	79 (71.2%)
Palatal petechiae	3 (8.3%)	16 (17.4)	20 (18.0%)
Abnormal chest X ray	7/20 (35.0%)**	40/46 (87.0%)*	49/61 (80.3%)*
Hepatomegaly		5 (5.4%)	7 (6.3%)
Splenomegaly	0 (0.0%)	3 (3.3%)	1 (0.9%)
ALC >10%	3/21 (14.3%)	9/51 (17.7%)	13/70 (18.6%)
Elevated ESR	10/21 (47.6%)	35/67 (53.2%)	30/63 (47.6%)
Elevated CRP	10/21 (47.6%)	33/68 (48.5%)	42/73 (57.5%)
ALF	4/18 (22.2%)	3/24 (12.5%)	12/33 (36.4%)

**IM**, infectious mononucleosis; **Non-IM**, patients without IM; **ALC**, atypical lymphocytes; **ESR**, erythrocyte sedimentation rate; **CRP**, C-reactive protein; **ALF**, abnormal liver function (alanine aminotransferase or aspartate aminotransferase levels >46 U/L). *P* < 0.05 between * and **.

### Clinical features of different antibody combinations

To understand the implication of different antibody combinations, five patterns were compared: group A [VCA-IgM+, VCA-IgG– and EBNA-IgG–]; group B [VCA-IgM+, VCA-IgG+ and EBNA-IgG+]; group C [VCA-IgM–, VCA-IgG+ and EBNA-IgG–]; group D [VCA-IgM–, VCA-IgG+ and EBNA-IgG+]; and group E [VCA-IgM–, VCA-IgG– and EBNA-IgG–]. The symptoms and physical signs seemed to be most severe in the patients of group A, especially an atypical lymphocyte count of >10%. The symptoms, physical signs and laboratory results in groups B and C were not more serious than in groups D or E. The pattern of group C [VCA-IgM–, VCA-IgG+ and EBNA-IgG–] did not have any specific clinical features (Table 8).

**Table 8:** Clinical features of different antibody combinations in EBV-positive patients (age ≥8 months)

**Clinical features**	**A (n = 17)**	**B (n = 8)**	**C (n = 13)**	**D (n = 112)**	**E (n = 121)**
Length of stay (days)	10.5 ± 2.9*	10.4 ± 4.9	9.0 ± 4.5	8.0 ± 2.9*	9.1 ± 4.0
Fever	17 (100.0%)	8 (100.0%)	12 (92.3%)	100 (89.3%)	106 (87.6%)
Cough	9 (52.9%)	8 (100.0%)	12 (92.3%)	79 (70.5%)	90 (74.4%)
Rash	4 (23.5%)	4 (50.0%)	0 (0.0%)	23 (20.5%)	19 (15.7%)
Lymphadenopathy	11 (64.7%)**	6 (75.0%)	5 (38.5%)	41 (36.6%)	35 (28.9%)*
Pharyngitis	16 (94.1%)	7 (87.5%)	10 (76.9%)	89 (79.5%)	87 (71.9%)
Palatal petechiae	9 (52.9%)**	2 (25.0%)	5 (38.5%)	20 (17.9%)*	24 (19.8%)
Abnormal chest X ray	8/9 (88.9%)	2/2 (100.0%)	3/5 (60.0%)	50/57 (87.7%)	53/65 (81.5%)
Bronchitis (X ray)	3/9 (33.3%)	1/2 (50.0%)	2/5 (40.0%)	32/57 (56.1%)	25/65 (38.5%)
Pneumonia (X ray)	5/9 (55.6%)	1/2 (50.0%)	1/5 (20.0%)	18/57 (31.6%)	28/65 (43.1%)
Hepatomegaly	5 (29.4%)	2 (25.0%)	3 (23.1%)	7 (6.3%)	11 (9.1%)
Splenomegaly	4 (23.5%)	0 (0.0%)	1 (7.7%)	7 (6.3%)	5 (4.1%)
ALC >10%	12/15 (80.0%)**	0/6 (0.0%)*	4/8 (50.0%)	14/66 (21.2%)*	18/77 (23.4%)*
Elevated ESR	8/14 (57.1%)	5/6 (83.3%)	2/10 (20.0%)	43/81 (53.1%)	31/66 (47.0%)
Elevated CRP	4/13 (30.8%)	5/6 (83.3%)	5/9 (55.6%)	42/83 (50.6%)	43/76 (56.6%)
ALF	9/13 (69.2%)	1/4 (25.0%)	2/6 (33.3%)	9/36 (25.0%)	15/38 (39.5%)

A: patients with the antibody combination of [VCA-IgM+, VCA-IgG– and EBNA-IgG–]. B: patients with the antibody combination of [VCA-IgM+, VCA-IgG+ and EBNA-IgG+]. C: patients with the antibody combination of [VCA-IgM–, VCA-IgG+ and EBNA-IgG–]. D: patients with the antibody combination of [VCA-IgM–, VCA-IgG+ and EBNA-IgG+]. E: patients with the antibody combination of [VCA-IgM–, VCA-IgG– and EBNA-IgG–]. **ESR**, erythrocyte sedimentation rate; **CRP**, C-reactive protein; **ALC**, atypical lymphocytes; **ALF**, abnormal liver function (alanine aminotransferase or aspartate aminotransferase levels >46 U/L). *P* < 0.05 between * and **.

## Discussion

We found that EBV was a common pathogen in these Chinese children with respiratory tract infections and that those aged 8–36 months had the highest risk of a primary EBV infection. The determination of immunoglobulins against specific antigens of EBV is recommended in pediatric patients [15,17]. VCA-IgM is generally designated as an indicator of recent primary infection. VCA and EBNA cause lifelong persistent IgG titers. The avidity of VCA-IgG antibodies detected is low in samples from patients with recent EBV infections and high in patients with a past infection or reactivation [10,17]. The combination of negative EBNA-IgG and low-avidity VCA-IgG has given a sensitivity and specificity of 100% [4].

In the present study, there were no differences between the age groups in the detection rates for VCA-IgM, EA-IgG and low-affinity VCA-IgG. The detection rates were the lowest among those aged 8–24 months and increased gradually with age. These two antibodies are the most frequent in this age group because of the persistence of maternal antibodies in children aged <8 months; after the maternal antibodies disappear, the infection rate increases gradually. Most maternal antibodies disappear at 4 months of age and are no longer detectable at 8 months [11]. To exclude the impact of maternal antibodies, patients were divided into two groups, with 8 months as the cutoff. Five patients had the combination of VCA-IgM+ with low-affinity VCA-IgG–, three of whom presented with a primary cytomegalovirus (CMV) infection. This is known to produce a false-positive result for VCA-IgM [18], so given the protective role played by maternal antibodies, we surmise that the positive result for VCA-IgM in these patients was induced by the CMV infection. The youngest infant with a primary EBV infection having the pattern of [VCA-IgM+, low affinity VCA-IgG+, EBNA-IgG+, VCA-IgG– and EA-IgG–] was only 1 month old, and this was probably caused by an intrauterine or intrapartum infection. Previous studies have shown that EBV might be present in cervical secretions, and EBV seems to be able to cross the placenta and to cause placental infections manifested by deciduitis and villitis [19].

The presence of VCA-IgG in the absence of VCA-IgM and EBNA-IgG antibodies makes classifying EBV infection more difficult. This serological picture can be seen in cases of past infection with EBNA-IgG showing loss or nonappearance of these antibodies, or in patients with primary EBV infections with the early disappearance or delayed onset of production of VCA-IgM. In this series, 13 patients (4.64%) were diagnosed with orphan VCA-IgG+. A study in 2009 found that the prevalence of the [VCA-IgG+, VCA IgM– and EBNA-1-IgG–] serological combination in children aged 1–10 years was 4.5%, and it was associated mainly with acute infection [20,21]. In the present study, patients with this serological pattern seemed to have more severe symptoms, physical signs and adverse laboratory results, especially an atypical lymphocyte count of >10% compared with the pattern [VCA-IgM–, VCA-IgG+ and EBNA-IgG–]. This could have been associated with an EBV primary infection causing IM. EBV-associated IM occurred in all age groups, with a peak incidence at 2–4 years in Hong Kong [22], which is similar to the result of our study (a peak incidence at 2–3 years).

The disease spectrum associated with a primary EBV infection is very diverse. In most studies published outside China, about 50% of children with an EBV infection develop IM [23], and the proportion of IM seen in our study was similar (40%) and much higher than other studies in China [24,25], where the proportion of IM in the disease spectrum was only 17.9%. However, our results show that EBV mainly caused respiratory tract infections. These results suggest that EBV is a common pathogen in respiratory tract infections as well as in IM. EBV seropositivity among children has a geographic variation: the age of primary infection is earlier in Asia and other developing countries than in Western countries. The infection rate of 2-year-old children exceeded 60.7% in Hong Kong in 2001 [12]. In our study, the positive rate among children aged 2–3 years was 64.5%. However, it was 84.2% among those aged ≥9 years, so there was no obvious decline in the infection rate.

## Conclusions

These Chinese infants aged <8 months could be infected by EBV and about 84% of children aged ≥9 years had serological evidence of EBV infection. In addition to causing IM, EBV was also a common pathogen in children with respiratory tract infections. The detection rate of EBV-related IM peaked in patients aged 2–3 years.

## Abbreviations

EBV: Epstein–Barr virus;CMV: Cytomegalovirus;IM: Infectious mononucleosis;VCA: Viral capsid antigen;EA: Early antigen;EBNA: Epstein–Barr virus nuclear antigen;IFA: Indirect immunofluorescence assay;IgG: Immunoglobulin G;IgM: Immunoglobulin M

## Competing interests

The authors declare that they have no competing interests.

## Authors’ contributions

HY collected the data and wrote the manuscript. WC and ZK discussed and reviewed the manuscript. DZ designed the manuscript and analyzed the data. All authors read and approved the final manuscript.
